# MicroRNA-Gene Association As a Prognostic Biomarker in Cancer Exposes Disease Mechanisms

**DOI:** 10.1371/journal.pcbi.1003351

**Published:** 2013-11-21

**Authors:** Rotem Ben-Hamo, Sol Efroni

**Affiliations:** The Mina and Everard Goodman Faculty of Life Science, Bar Ilan University, Ramat-Gan, Israel; Institute for Systems Biology, United States of America

## Abstract

The transcriptional networks that regulate gene expression and modifications to this network are at the core of the cancer phenotype. MicroRNAs, a well-studied species of small non-coding RNA molecules, have been shown to have a central role in regulating gene expression as part of this transcriptional network. Further, microRNA deregulation is associated with cancer development and with tumor progression. Glioblastoma Multiform (GBM) is the most common, aggressive and malignant primary tumor of the brain and is associated with one of the worst 5-year survival rates among all human cancers. To study the transcriptional network and its modifications in GBM, we utilized gene expression, microRNA sequencing, whole genome sequencing and clinical data from hundreds of patients from different datasets. Using these data and a novel microRNA-gene association approach we introduce, we have identified unique microRNAs and their associated genes. This unique behavior is composed of the ability of the quantifiable association of the microRNA and the gene expression levels, which we show stratify patients into clinical subgroups of high statistical significance. Importantly, this stratification goes unobserved by other methods and is not affiliated by other subsets or phenotypes within the data. To investigate the robustness of the introduced approach, we demonstrate, in unrelated datasets, robustness of findings. Among the set of identified microRNA-gene associations, we closely study the example of MAF and hsa-miR-330-3p, and show how their co-behavior stratifies patients into prognosis clinical groups and how whole genome sequences tells us more about a specific genomic variation as a possible basis for patient variances. We argue that these identified associations may indicate previously unexplored specific disease control mechanisms and may be used as basis for further study and for possible therapeutic intervention.

## Author Summary

Despite major progress and improved understanding of Glioblastoma Multiforme, the disease is still associated with poor prognosis. The identification of genomic regulatory mechanisms, their affiliation with clinical outcome and the association between specific modifications in genome sequence that can explain gain and loss of such regulatory activity, combine to suggest specific disease mechanisms and possible means of intervention in the course of the disease. We report here a method and its implementation in exposing possible regulatory mechanisms in GBM. At the core of this method is the employment of associations between micro RNAs and genes as a quantifiable metric. Identification of these associations and their affiliation with clinical features, combined with the availability of whole genome sequences, brings forward specific micro RNAs and their associated genes. Affiliation of specific genomic sequences with clinical outcome thus translates personal genomics into tumor relevant decision-making.

## Introduction

Micro RNAs (miRs) are small, endogenous non-coding RNA molecules that control gene expression by inhibiting translation or inducing cleavage of target mRNAs. The role of miRs as key regulators of a wide variety of fundamental cellular processes, such as proliferation, apoptosis, differentiation, motility, invasiveness and more, is increasingly recognized in almost all aspects of biology and biomedicine [1]. MiRs are aberrantly expressed in cancer tissues and a connection between deregulated miRs and the inhibition of tumor suppressor genes in cancer is well established [1], [2]. Several studies have shown potential use for miR-based therapy in cancer [3]–[5]. An example is the use of anti-miR-21 in breast cancer, which led to the suppression of both cell growth in vitro and tumor growth in vivo [6]. The potential of miRs to act both as therapeutic agents and as disease biomarkers places this family of molecules at the forefront of biomedical interest [7].

Several thousand human genes are potential targets for regulation by several hundred miRs encoded in the genome. The common hypothesis is that miRs down-regulate protein expression by inhibiting target mRNA translation or by increasing mRNA degradation [8]. Recent evidence also suggests that they may up-regulate protein expression by up-regulating transcription [9]. Both up-regulation and down-regulation of RNA levels by miRs would result in a quantifiable measurement - the correlation between the expression levels of the miR and its target mRNA.

Glioblastoma Multiforme (GBM) is the most common, aggressive and malignant primary tumor of the brain and is associated with one of the worst 5-year survival rates among all human cancers [10]. Advances in treatment for newly-diagnosed GBM have led to the current 5-year survival rates of 9.8%. Despite therapy, once GBM progresses, the outcome is uniformly fatal, with median overall survival historically less than 30 weeks [11].

One of the most comprehensive efforts at molecular characterization of cancer in general and Glioblastoma Multiforme in particular is The Cancer Genome Atlas (TCGA) [12]. The types of data provided through TCGA for over 370 GBM patients are: clinical, expression abundance through microarrays, microRNA expression levels and whole genome sequencing for a subgroup of patients.

Here, by studying the entire set of pairs of genes and miRs across the different platforms and by applying feature-selection algorithms on miR and gene co-expression, we were able to classify patients into two groups, one displaying valid association of the target RNA by the miR, and the other showing no such association. This association is quantified by measuring the correlations between miRs and target RNAs and is tagged ‘valid’ when the correlation is significantly negative. To see if this metric affiliates with disease outcome, we performed Kaplan-Meier (KM) survival analysis on these results. Surprisingly, we identified 7,509 pairs in which the pair-based stratification was associated with survival outcome. To correct for multiple hypotheses, we used Bonferroni correction to adjust the KM p-values. This procedure left us with a subset of pairs that presented both a valid and a prognosis-informative correlation. We then used the miRecord comparison survey [13] to identify possible binding sites for the miR through which gene expression could be regulated. Further, to avoid classification otherwise simply based on either miR or gene expression levels, we filtered out pairs in which the gene or miR showed significant lower levels of expression in one group compared to the other.

The 17 miR-gene pairs identified included four traditional and 13 non-traditional (we use the term ‘non-traditional pair’ for a miR-gene pair in which the miR binding site does not start on the second position). The resulting set of miR-gene pairs therefore combines the following features: a possible binding site for the miR within the gene, a strong negative correlation in one of the clinical groups, and a highly significant patient stratification to disease outcome.

The work presented here demonstrates how association between miR and mRNA levels provides a biomarker by which patients can be stratified to disease outcome in a highly significant manner. This association may provide insight into the mechanism of the disease. By tagging these associations and by affiliating them with clinical outcome, the presented method provides an agenda for a signature based research into tumor cell network mechanisms.

## Results

**Figure 1:**
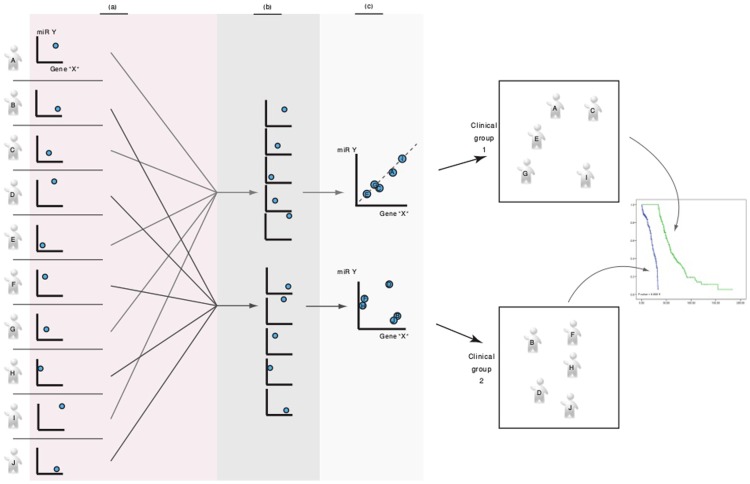
Workflow diagram. Using standard feature selection algorithms (see Materials and Methods), patients are stratified into two groups. One group is populated by samples with a demonstrated correlation between the miR and the gene. The other group is populated with the samples that together show absent correlation. The algorithm populates these gene-miR correlation groups iteratively; a patient is added to a group according to its contribution to the correlation. Once groups are formed, we quantify their clinical relevance through a Kaplan-Meier survival analysis. Bonferroni correction for multiple hypotheses is performed to choose a significant set of miR-gene pairs. These pairs are then tested in whole genome sequencing data to determine gene-miR binding sites and to identify tumor related modifications in these genomic sites.

We used machine learning algorithm to affiliate clinical groups with combinations of the expression levels of roughly 23,000 genes and 1,510 miRs (see Materials and Methods). Using these methods, detailed below and further in the Methods section, we identified a set of miR-gene pairs with a specific behavior of the expression levels correlations. These correlations were used to stratify patients into clinical phenotypes. To determine these stratification-informative correlations, we scanned for correlations between pairs of miRs and genes. We chose for correlations that were present only in a subset of patients and were absent from the complementary set. That is, for a miR-gene pair to be considered relevant, the correlation between the miR and the gene must be significant within one patient subgroup but insignificant within the other (see Figure 1). The assumptions underlying this stratification method are: (1) miRs control a genes' mRNA abundance, (2) specific regulation of a gene by a miR is prone to changes with accumulated genomic aberrations. (3) Such an accumulation of genomic aberrations may be affiliated with patient subgroups.

Computationally, these differences in regulation would lead to differences in miR-gene associations. These association allow us to provide two patient subsets, one in which the control mechanism and hence the computational association and calculated correlation is strong and another in which the control mechanism, the miR-gene association and the miR-gene correlation are weak. Where regulation is strong, expression data will show a negative correlation between the miR and its cognate gene. Where regulation is weak, there will be no miR control over gene expression and expression levels of the miR and its cognate gene will not show the expected correlation.

Kaplan-Meier (KM) survival analysis, a well-established quantifiable metric for stratifying patients according to disease outcome [14], is often used in clinical and basic research to identify specific modifiable targets that may associate with survival rates. In GBM datasets, owing to disease course, other available patient relevant data are often either absent or uninformative (such as disease stage, which is often unimodal across patients; pharmaceutical regimen, which we discussed earlier or environmental parameters, where no significant association has been determined), and leaves disease outcome as the only strong phenotype available. We used this disease outcome information, by performing KM survival analysis within patient groups and stratified groups through miR-gene pairing. Then, to screen for unique miR-gene pairs, we employed feature-selection analysis (described in Methods) and selected for pairs whose miR-gene correlation metric is also relevant in stratifying prognosis. Such prognosis groups are groups in which a miR is associated with its cognate gene in one subset of patients and not in the complementing set. One group is affiliated with good prognosis and the other with poor prognosis. These calculations ultimately lead to a set of p-values over a large cohort of tested pairs, and thus require adjustment for multiple hypotheses. We applied Bonferroni correction to the KM p-values, which led us to include only pairs with p-values under 6.6×10^−6^ (0.05/7509 pairs). Twenty-six miR-gene pairs maintained a significant p-value after this adjustment. Additional filtering was then implemented to negatively select pairs in which one of the components (either miR or gene) displayed any meaningful association with one of the groups. This filtering has been added to avoid cases in which the gene or the miR displayed significant lower expression values in one group, which might render any possible association as useless (assuming no regulation could be made when one of the participants is absent) Filtering resulted in a set of seventeen pairs.

Once we had identified the relevant miR-gene pairs, we set out to identify specific genetic modifications that could explain some of the (dys)function of these control mechanisms. To identify relevant genomic regions we used miRecords [13], a set of tools for the predictions of target sites for each of the miRs on its cognate, paired, gene. MiRecords summarizes predicted targets from 11 different prediction tools. This extensive filtering procedure left us with 16 miR-gene pairs (shown in Table 1). Here, we follow one of the pairs – the gene MAF and the miR hsa-miR-330p as an example, to demonstrate some of the biological insight mined by joining in behavior at the miR and gene expression levels with sequence based information.

**Table 1:** Prediction of miR binding sites using miRecords identified 24 gene-miR pairs with binding sites.

microRNA	Gene	KM Pval	DIANA	Micro Inspector	miRanda	Mir Target2	mi Target	NB miRTar	PicTar	PITA	RNA 22	RNA hybrid	TargetScan
hsa-miR-330-3p	MAF	0.000000003			✓					✓	✓	✓	
hsa-miR-768-5p	OCLN	0.0000001								✓	✓	✓	
hsa-miR-769-5p	SLC24A6	0.0000004								✓	✓	✓	
hsa-miR-202	SOCS5	0.0000007								✓	✓	✓	
hsa-miR-768-5p	DYRK1A	0.000002			✓					✓		✓	
hsa-miR-660	BIN1	0.000003								✓	✓	✓	
hsa-miR-330-3P	CYLD	0.000005			✓					✓		✓	
hsa-miR-500	ANKHD1	0.0000008									✓	✓	
hsa-miR-7	RPN2	0.000003									✓	✓	
hsa-miR-365	FADS1	0.000004								✓		✓	
hsa-miR-301	GPR68	0.000005								✓		✓	
hsa-miR-128a	MAP3K14	0.000003										✓	
hsa-miR-489	MOSC2	0.000004										✓	
hsa-miR-556	TARS2	0.000004										✓	
hsa-miR-373	TBCE	0.000005										✓	
hsa-miR-126	GPN3	0.000005										✓	

We considered bindings site as those predicted by at least one prediction. The table shows the 24 pairs, their corresponding Kaplan-Meier p-values and the 11 utilities used by the miRecords algorithm. The number of predicting algorithms per miR-gene pair sorts the table.

**Figure 2:**
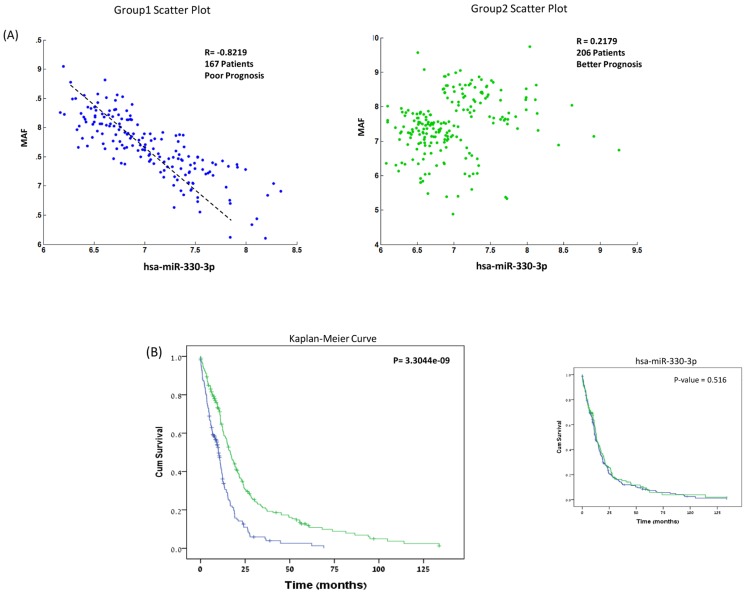
373 GBM patients were stratified using the described method and analyzed for the association between miR-gene behavior and prognosis. (A) The set of patients is assigned to different cohorts according to contribution, or lack of, to the correlation metric between MAF and hsa-miR-330-3p (text and Figure 1). The left hand side panel is composed of the pool of samples that merge to display a strong (negative) correlation, while the right hand side panel displays the pool of samples that merge to give no significant correlation. (B) Kaplan-Meier survival curves of the groups that emerged from the analysis. Group 1 (blue line) has lower survival rates (and a significant negative correlation between the miR and the gene), and Group2 (green line) has higher survival rates. The right-hand panel in (B) also shows how hsa-miR-330-3p itself, without its use in the metric, does not provide any significant stratification.

Figure 2 provides the case of MAF and hsa-miR-330-3p as an example for classification into groups. In Group 1, we see a highly significant correlation between miR-330-3p and MAF (R score = −0.8219). This group displays poor prognosis. In contrast, the correlation between the gene and miR expression levels in Group 2 is close to zero, together with significantly better (p-value = 3.3044e-09) prognosis. This miR-gene correlation, associated with a more violent form of tumor behavior, may be the result of an acquired tumor network mechanism, which, as we see below, may be the result of specific base modifications in the poor prognosis group.

**Figure 3:**
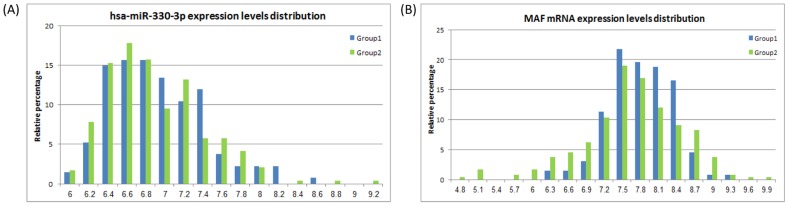
Gene expression levels of hsa-miR-330-3p and MAF in Group 1 and Group 2. The figure displays no significant change in expression levels between the groups, despite the presentation of a specific allele in the miR promoter region in Group 2.

To identify possible genomic modifications, the result of which could be this acquired association, and which may explain lack and gain of regulation mechanisms, we examined whole genome sequence (WGS) data, which is available for 19 of the patients in this study. We applied the classification detailed above, to affiliate these 19 patients with Group 1 or 2 described above. That is, patients were tagged as either Group 1 or Group 2 according to their miR-gene correlation coefficients described earlier. Of the 19 patients with an available WGS, eleven were classified as Group 1 and eight were classified as Group 2. We first set out to identify whether the patients had any somatic mutations in the coding regions for the gene and/or for the miR. No mutations were found in these regions, neither for MAF, nor for hsa-miR-330-3p, for any of the patients. Nevertheless, in a manner that supports our association hypothesis, the set of 11 patients from Group1 all shared a SNP (rs147260403) G/T in their miR promoter region. This SNP has been absent from the sequences of the 8 patients from Group 2. Table S1 contains the patient's barcode and genotype association. In a very interesting manner, display, or lack of display of this allele in the miR promoter region did not associate with lower expression levels of hsa-miR-330-3p, as shown in Figure 3.

**Figure 4:**
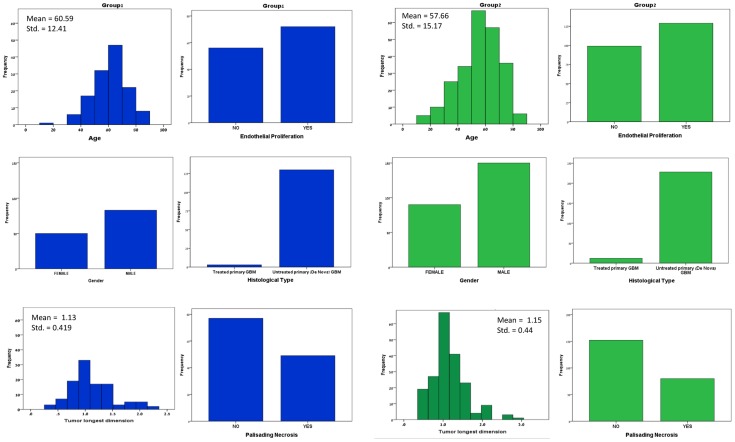
Distribution of clinical features in the two groups stratified by the miR-gene metric according to the following features: Age, Gender, Tumor longest dimension, endothelial proliferation, Histological type and palisading necrosis. The figure demonstrates that the two groups display very similar clinical features.

Often, molecular markers do not reveal novel mechanism but rather hide sub-clinical states which are displayed as molecular markers. To avoid such bias in our study, we set out to confirm that the stratification performed here is indeed based solely on the presented metric and is not a recapitulation of clinical variables. For this end, we performed additional analysis on possible links between the clinical measurements assessed and the groups that emerged. This analysis revealed that the classification was indeed a consequence of miR-Gene correlation and not a rearrangement of well-known clinical features, demographic features or disease history. Figure 4 shows clinical measurement distributions in the two groups.

**Figure 5:**
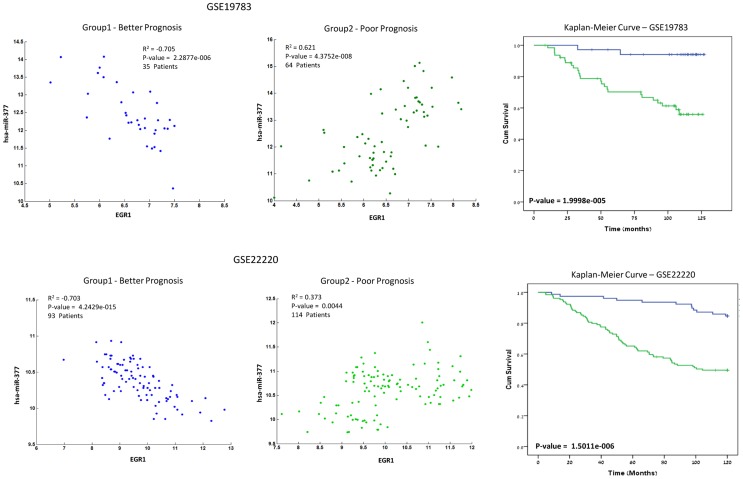
Breast cancer patients from two independent datasets were stratified using the described method and analyzed for the association between miR-gene behavior and prognosis. In a manner similar to the one described in Figure 2, the set of patients is stratified, this time by the correlation metric between EGR1 and hsa-miR-377. Again, as in Figure 2, the two panels give the samples that follow (left) a strong correlation, and (right), no correlation. Then, a Kaplan-Meier survival analyses is conducted on the two groups, and demonstrates how Group 1 (blue line) has higher survival rates (and a significant negative correlation between hsa-miR-377 and EGR1), and Group 2 (green line) has lower survival rates.

Over the past few years, robustness proved to be highly important feature in any computational analysis, and conclusions are much strengthened when they are supported by additional, independent datasets. While TCGA is the only dataset today that provides a combination of gene-expression, microRNA expression, and clinical data from GBM patients, other datasets do provide similar data for breast cancer. Hence, we applied the pipeline detailed above on two unrelated breast cancer datasets (GSE19783 [15], GSE22220 [16]) that combine gene-expression, microRNA expression and clinical data. As previously specified, we identified a set of miR-gene pairs with expression levels correlated in a way that stratifies patients into clinical phenotypes in the two datasets. This analysis produced 2,450 pairs in the first dataset and 2,745 in the second, which were shown to have significant correlation in only one group, and where these groups stratified prognosis (p-value<0.05). Bonferroni correction was applied to leave 15 pairs in the first dataset (GSE19783) and 6 pairs in the second dataset (GSE22220). Four pairs overlapped in both datasets and are presented in Table 2. Those four pairs were found to share consistent behavior across the two datasets. Furthermore, the four genes and miRs that were found did not stratify prognosis by themselves as can be seen in Figure S2 and Figure S3. In addition, we performed a genome wide, single gene and single miR based survival analysis in order to identify single gene or microRNA that stratify prognosis in both breast cancer datasets that were tested. In the single miRNA analysis three microRNA were found to be significant in both datasets (hsa-miR-105, hsa-miR-190, hsa-miR-433), however, in the single gene analysis we could not find even one gene that stratify prognosis robustly in both datasets. We believe that the pipeline presented here can overcome the problems of different batches of experiments and can retrieve robust and consistent results. The most significant pair in these four samples was Early Growth Gene 1 (EGR1), which was paired with hsa-miR-377 (Figure 5). The classification we identified suggests that negative correlation between EGR1 and hsa-miR-337 may relate with higher survival rates while a positive correlation relates with poor survival rates. The transcription factor EGR1, is induced during G0–G1 transition of the cell cycle in a variety of cell lines upon mitogenic stimulation. Pervious observations suggested that the EGR genes were involved in controlling cellular proliferation [17]–[19]. EGR1 was found to be up regulated in a variety of cancer including breast cancer. In addition, it has been linked with transforming growth, multidrug resistance, proliferation and migration [20]. This behavior may explain some of the results shown here. Negative correlation between miR-337 and EGR1 may provide a regulation mechanism to downregulate EGR1 activity, which translates into better prognosis. In contrast, lack of this association and lack of this mechanism may lead to lower survival rates.

**Table 2:** The list of stratifying pairs in the two unrelated breast cancer datasets.

Data Set ID	microRNA	Gene	KM Pval	DIANA	Micro Inspector	miRanda	Mir Target2	mi Target	NB miRTar	PicTar	PITA	RNA 22	RNA hybrid	Target Scan
GSE 19783	hsa-miR-889	HS3ST4	1.9E-06											
GSE 19783	hsa-miR-885-5p	LEPREL2	2.8E-06										+	
GSE 19783	hsa-miR-195*	NEK11	3.1E-06											
GSE 19783	hsa-miR-371-5p	PSME1	3.8E-06										+	
GSE 19783	hsa-miR-141*	VAT1	4E-06											
GSE 19783	hsa-miR-301b	BAP1	4.1E-06								+		+	
GSE 19783	hsa-miR-185	KATNA1	4.7E-06			+							+	
GSE 19783	hsa-miR-187*	HECTD2	5.1E-06											
GSE 19783	hsa-miR-92a	HIVEP1	5.3E-06								+		+	+
GSE 19783	hsa-miR-612	RAB3B	5.4E-06								+		+	
GSE 19783	hsa-miR-324-3p	MAP3K9	5.6E-06								+		+	
GSE 19783	has-miR-16	VAT1	1E-05								+		+	+
GSE 19783	has-miR-377	EGR1	1.99E-05			+					+		+	+
GSE 19783	has-miR-671-5p	NEK11	2E-02			+					+		+	+
GSE 19783	has-miR-218	HOXB3	2E-05			+		+			+		+	+
GSE 22220	hsa-miR-33	EGR1	9E-07								+		+	
GSE 22220	hsa-miR-193a	FLJ33641	1E-06								+		+	
GSE 22220	has-miR-377	EGR1	1.50E-06			+					+		+	+
GSE 22220	has-miR-218	HOXB3	1.2E-05			+		+			+		+	+
GSE 22220	has-miR-16	VAT1	1.5E-05								+		+	+
GSE 22220	has-miR-671-5p	NEK11	1.62E-05			+					+		+	+

The table further provides information on predicted binding within the pairs.

## Discussion

Results presented here join other findings that support a critical core for miR regulation in cancer in general and in GBM in particular. The computational procedure we apply here, of using feature-selection algorithms to select for correlation and non-correlation of miR levels and gene expression levels, we identify subgroups with distinct behaviors. This behavior of these clinical groups is unseen by other computational approaches. The statistically highly significant affiliation between emerging groups and patient outcome suggests that these identified associations may indicate modifications to critical regulatory mechanisms in the disease. Future therapeutic modifications may lead to improve survival rates.

As shown in the above detailed example, the presence of association between MAF and hsa-miR-330-3p is linked to a decrease in overall survival rates. Interestingly, DNA sequence analyses shows allelic behavior of a specific SNP in the hsa-miR-330-3p is presented in conjunction with absent miR-gene association and with overall survival rate increasing dramatically (p-value = 1×10^−8^).

The association between MAF and hsa-miR-330-3p in only one subgroup of the patients, in addition to the better prognosis found in the uncorrelated, genotype-modified group, calls for the gene and the miR to be studied together as possible novel therapeutic targets.

In addition, validation on two independent breast cancer datasets strengthened the approach by showing overlap (i.e. robustness) in results. Taken together, these findings suggest such methods as basis for biomarkers to separate patient groups and uncover disease relevant regulation candidates.

In conclusion, the identification of genomic regulatory mechanisms, their affiliation with clinical outcome and the association between specific modifications in genome sequence that can explain gain and loss of such regulatory activity, combine to suggest specific disease mechanisms and possible means of intervention in the course of the disease. This discovery has been made possible by employing regulation as a quantifiable metric, combined with the availability of whole genome sequences. While in practice, such detailed knowledge of a patient's genomic data may only be available through personal genomics [21], progress in the field and decline in costs of molecular characterization together place such implementations at center stage.

## Materials and Methods

### TCGA

All data were obtained from The Cancer Genome Atlas (TCGA) database, available at http://cancergenome.nih.gov/. This dataset comprises molecular characterizations from over 370 glioblastoma patients. For each patient, the database provides gene expression, miR expression values and whole genome sequencing data for 19 patients in this study. In addition, the following clinical data variables were recorded for each patient: age, gender, vital status and chemotherapy status.

Gene expression was quantified using an Affymetrix HT Human Genome U133 Array Plate Set. The expression data were normalized by Quantile normalization to produce RMA levels [22].

### Validation set 1

Validation set 1, from Enerly et al. [15] is composed of gene expression, microRNA expression and clinical information from 99 breast cancer patients (GEO accession [GSE19783]). Gene-expression was quantified using Agilent whole genome microarray 4X44K, and miRNA expression was quantified using Agilent Human miRNA Microarray 2.0.

### Validation set 2

Validation set 2, from Buffa et al. [16] is composed of gene expression, microRNA expression and clinical information from 99 breast cancer patients (GEO accession [GSE22220]). Gene-expression was quantified using Illumina humanRef-8 v1.0 expression beadchip, and miRNA expression was quantified using Illumina Human v1 MicroRNA expression beadchip.

### Feature-selection

The expression levels of all pairs were obtained from the TCGA database and feature-selection algorithms were applied to identify a subgroup with significant negative correlation between the expression of the miR and its predicted gene target. In each iteration the algorithm added one patient's expression data to the analysis; if this data point diminished the correlation value and the significance, then the algorithm excluded the patient from the group. The algorithm iterated across groups until the highest value was reached. Only the miR-gene pairs that produced a subset of patients with significant negative correlation and insignificant correlation in the remaining set were selected.

### Survival analysis

Kaplan-Meier survival analysis was performed on all the pairs extracted from the feature-selection analysis [23], through clinical data (Vital Status), to determine the power of a pair for survival stratification. This analysis was done to identify pairs that could stratify prognosis on the basis of the presence or absence of a correlation.

Bonferroni correction for multiple comparisons was applied to the p-value so only those pairs with p-value<6.6×10^−6^ were extracted. Twenty-six pairs were found to be significant in prognosis stratification after the adjustment.

### MicroRNA binding site prediction

Using the miRecord comparison survey [13], we predicted microRNA binding sites on the 26 miR-gene pairs that were correlated in only one subset of patients and significant in prognosis stratification. miRecord is an integrated resource for miR-target interaction prediction that combines predictions from 11 existing programs including TargetScan [24]/TargetScanS [25], PicTar [26], miRanda [27], DIANA-microT [28] and MicroInspector [29]. We identified 24 genes out of the 26, by at least one program, as having a possible binding site for their corresponding miR. Table 1 details these results.

### Algorithm (pseudo code)

1. For every gene

1.1 For every microRNA

1.1.1 Randomly choose three patients - Group1

1.1.2 Corr = correlation (Group1_microRNA, Group1_gene-expression);

1.1.3 For k = 1:(number_of_patients - 3)

1.1.3.1 Add a patient to Group1

1.1.3.2 Tmp_corr = correlation (Group_microRNA, Group_gene-expression);

1.1.3.3 If (abs (Corr)>abs (Tmp_corr))

1.1.3.3.1 Leave patient at Group2

1.1.3.4 Else

1.1.3.4.1 Add patient to Group1

1.1.3.5 End

1.1.4 End

1.1.5 If one group is significant and the other is insignificant

1.1.5.1 Survival_pval = logrank (Vital_Status, Groups);

1.1.6 End

1.2 End

2. End

### False discovery rate analysis

With the increase in genome-wide sequencing and high-throughput technologies, the analysis of large data sets has moved into the mainstream of computational biology. It is often the case that thousands of features in a genome-wide data set are tested against some null hypothesis, where a number of features are expected to be significant. False discovery rate (FDR) [30] is a statistical method aimed at correcting for multiple hypothesis comparisons, much as the Bonferroni correction does. Here, to determine the FDR for the 26 miR-gene pairs described above, we performed FDR analysis on the dataset.

The FDR algorithm iteratively randomizes the survival rates 1000 times to identify miR-gene pairs that stratify prognosis according to the randomized survival, thus mimicking the results for a random non-disease-related state. The results revealed that out of 1000 iterations across more than 100,000 pairs, the highest number of pairs that sustained Bonfferoni correction was six. Figure S1 shows the distribution of the results. Therefore, the FDR for finding 26 significant miR-Gene pairs is <0.001, which reinforces the hypothesis that the results shown here provide insight into the mechanism of the disease through miR-Gene association.

### Algorithm sensitivity analysis

Sensitivity analysis was performed in order to evaluate the algorithm performance when the data is scrambled. Our feature selection algorithm starts by randomly choosing three patients and then iterates across all patients in order to 1) stratify patients by their miR-gene correlation status and 2) evaluate the effect these groups have on the patient's survival status. In order to evaluate any possible outcome, which may the result of different initial conditions, i.e., a different initial 3-sample set; we randomly choose 1000 pairs of miR-gene and calculated the entire set of three possible combinations to start from. Figure S1 demonstrates the average and standard deviation for all combinations in the 1000 pairs selected in addition to the 17 pairs identified in the analysis. These results highlight, once again, the robustness of the algorithm. The initial selection of three patients eventually does not affect final results.

## Supporting Information

Figure S1 The figure shows a sensitivity analysis, evaluating the algorithm performance over different initial conditions. An exhaustive search over possible initial 3-sample choice is made. As the feature selection algorithm starts by randomly choosing three patients and then iterating across all patients, this sensitivity analyses measures the effect of the initial 3-sample choice over a random set of 1000 pairs, as well as the pairs discussed in the paper. For this set of 1000 pairs, calculated sensitivity determining the difference in results that stems from initial choice. This figure gives the average and standard deviation of all combinations in the random 1000 pairs selected. As the figure shows, these initial conditions do not significantly affect the final results.
(TIF)

Figure S2 Kaplan-Meier survival curves of the four genes that emerged from the analysis in the tow breast cancer datasets that were analyzed. Group 1 (blue line) indicates on lower expression levels, and Group2 (green line) indicates on higher expression levels.
(PPTX)

Figure S3 Kaplan-Meier survival curves of the four microRNAs that emerged from the analysis in the tow breast cancer datasets that were analyzed. Group 1 (blue line) indicates on lower expression levels, and Group2 (green line) indicates on higher expression levels.
(PPTX)

Table S1 The table presents patients' genotype and their barcode in relevance to the identified SNP discussed in the text. As the table demonstrates, there is a perfect overlap between the miR-gene group affiliation and the genotype.
(DOCX)
