# Supplementary figures and images for: MicroRNA-Gene Association As a Prognostic Biomarker in Cancer Exposes Disease Mechanisms
Source: PLoS Comput Biol. 2013 Nov 21;9(11):e1003351. doi: 10.1371/journal.pcbi.1003351 (PMC3836703; doi:10.1371/journal.pcbi.1003351)

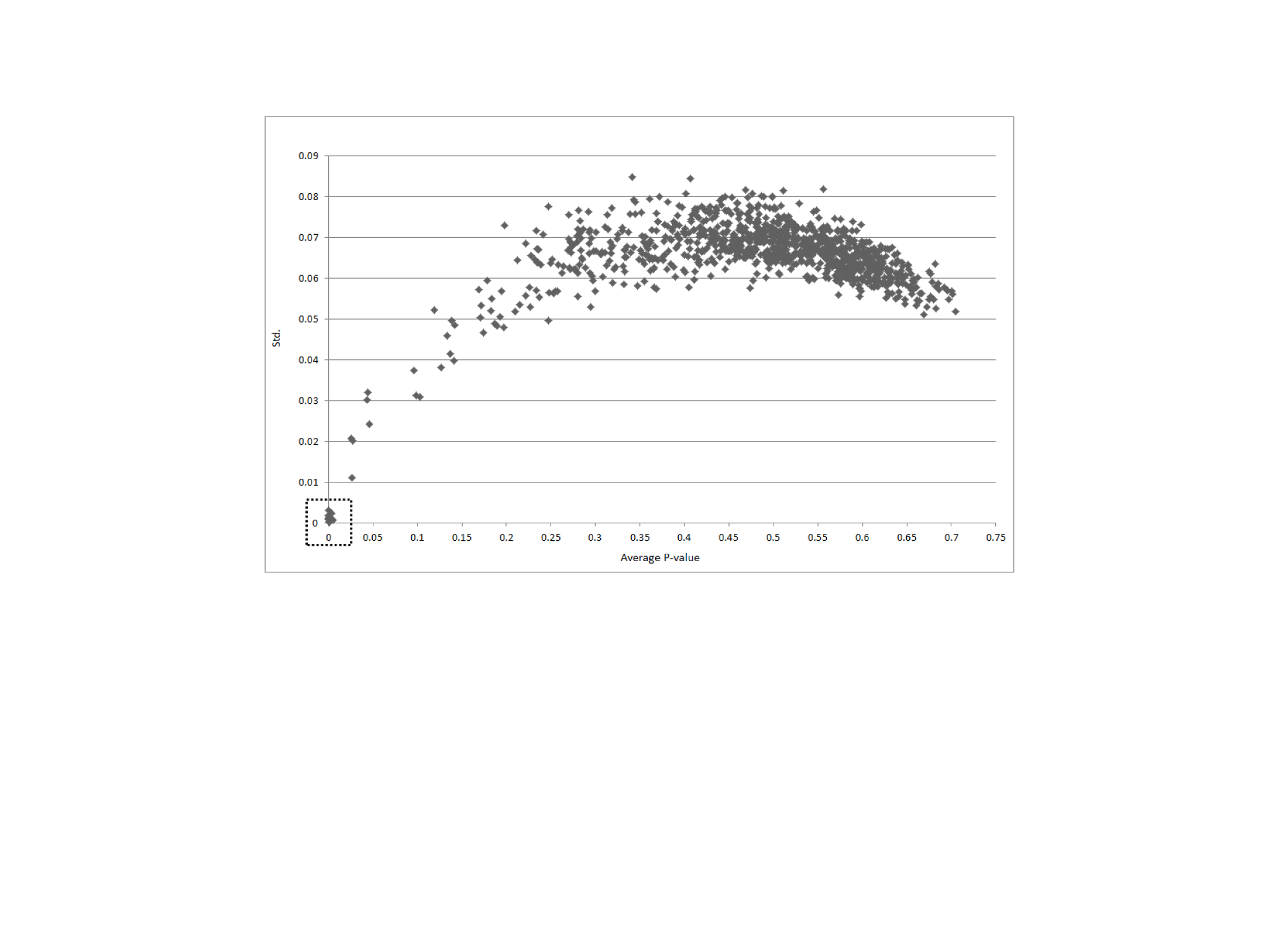

Supplement: Figure S1 — The figure shows a sensitivity analysis, evaluating the algorithm performance over different initial conditions. An exhaustive search over possible initial 3-sample choice is made. As the feature selection algorithm starts by randomly choosing three patients and then iterating across all patients, this sensitivity analyses measures the effect of the initial 3-sample choice over a random set of 1000 pairs, as well as the pairs discussed in the paper. For this set of 1000 pairs, calculated sensitivity determining the difference in results that stems from initial choice. This figure gives the average and standard deviation of all combinations in the random 1000 pairs selected. As the figure shows, these initial conditions do not significantly affect the final results. (TIF) [file pcbi.1003351.s001.tif]

## Slide 1
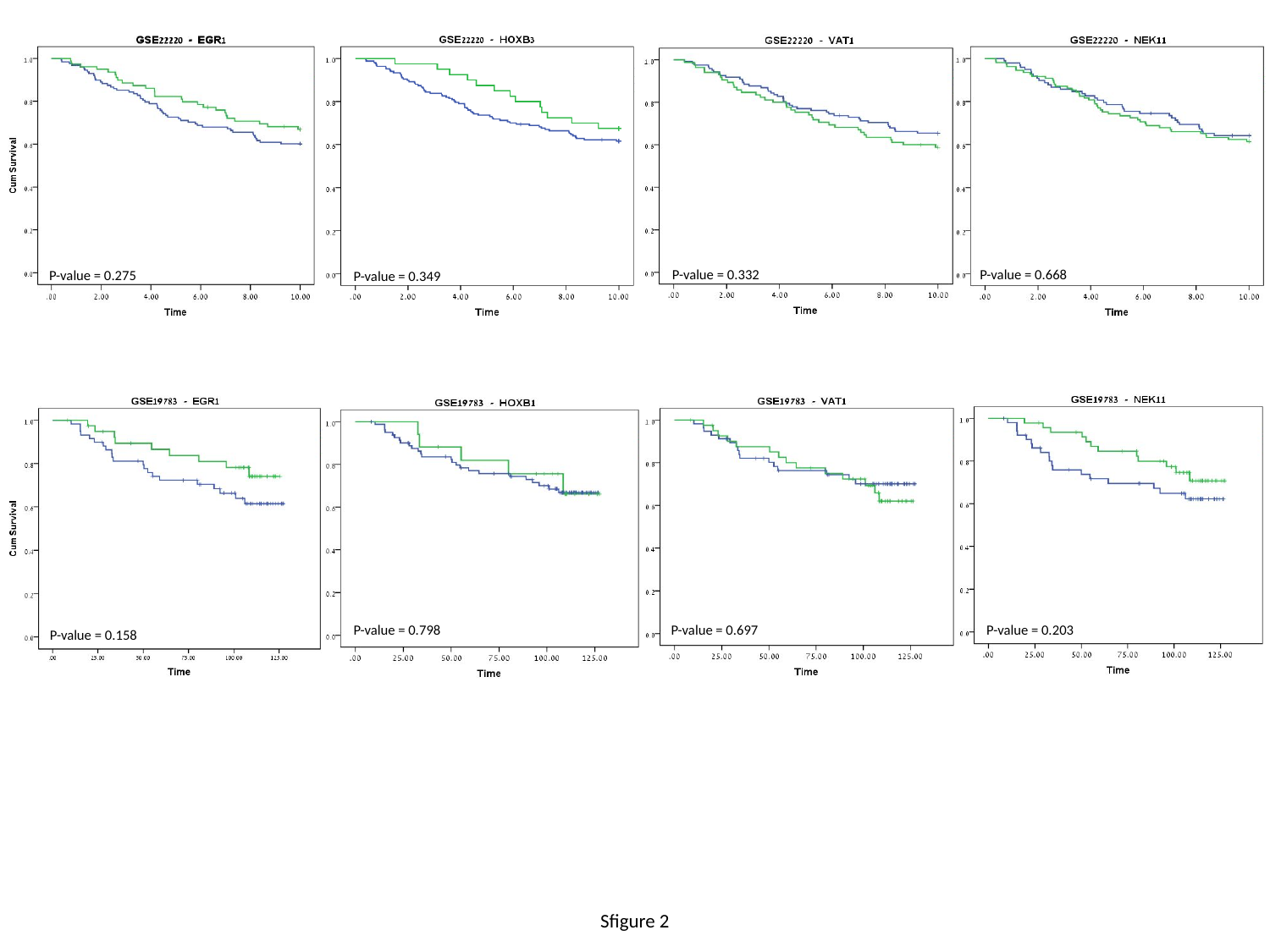

P-value = 0.332
P-value = 0.275
P-value = 0.349
P-value = 0.668
P-value = 0.203
P-value = 0.158
P-value = 0.798
P-value = 0.697
Sfigure 2

Supplement: Figure S2 — Kaplan-Meier survival curves of the four genes that emerged from the analysis in the tow breast cancer datasets that were analyzed. Group 1 (blue line) indicates on lower expression levels, and Group2 (green line) indicates on higher expression levels. (PPTX) [file pcbi.1003351.s002.pptx]

## Slide 1
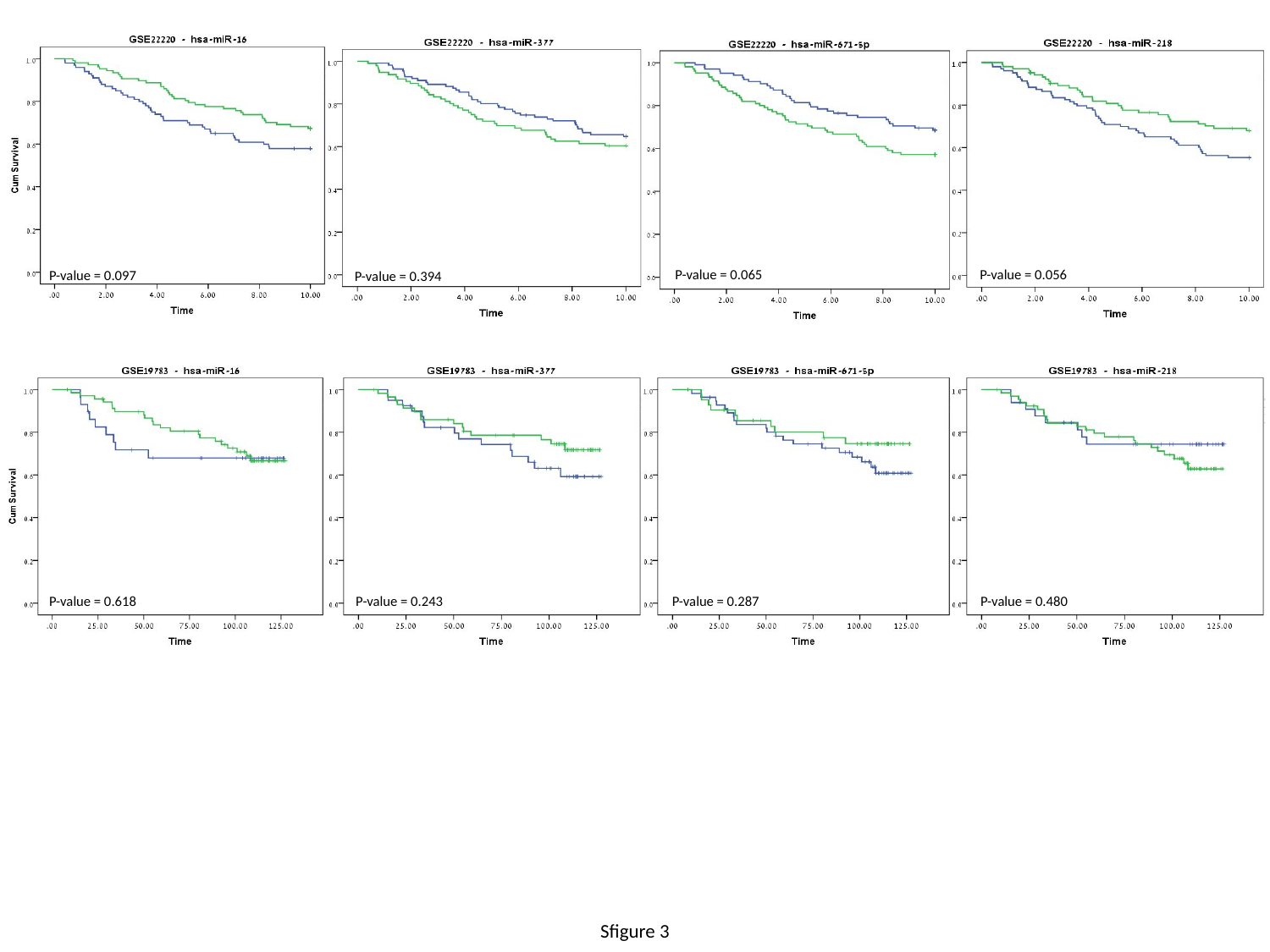

P-value = 0.394
P-value = 0.056
P-value = 0.065
P-value = 0.097
P-value = 0.618
P-value = 0.480
P-value = 0.243
P-value = 0.287
Sfigure 3

Supplement: Figure S3 — Kaplan-Meier survival curves of the four microRNAs that emerged from the analysis in the tow breast cancer datasets that were analyzed. Group 1 (blue line) indicates on lower expression levels, and Group2 (green line) indicates on higher expression levels. (PPTX) [file pcbi.1003351.s003.pptx]
