# Supplementary material for: MicroRNA-Gene Association As a Prognostic Biomarker in Cancer Exposes Disease Mechanisms
Source: PLoS Comput Biol. 2013 Nov 21;9(11):e1003351. doi: 10.1371/journal.pcbi.1003351 (PMC3836703; doi:10.1371/journal.pcbi.1003351)
Supplement: Table S1 — The table presents patients' genotype and their barcode in relevance to the identified SNP discussed in the text. As the table demonstrates, there is a perfect overlap between the miR-gene group affiliation and the genotype. (DOCX) [file pcbi.1003351.s004.docx]

Table S1

| Patient Barcode | miR-Gene group affiliation | rs147260403 |
| --- | --- | --- |
| TCGA-06-0155 | 1 | + |
| TCGA-06-0877 | 1 | + |
| TCGA-06-1086 | 1 | + |
| TCGA-14-0786 | 1 | + |
| TCGA-26-1438 | 1 | + |
| TCGA-14-1401 | 1 | + |
| TCGA-06-0208 | 1 | + |
| TCGA-06-0152 | 1 | + |
| TCGA-06-0185 | 1 | + |
| TCGA-06-0188 | 1 | + |
| TCGA-06-0648 | 1 | + |
| TCGA-06-0214 | 2 | - |
| TCGA-06-0881 | 2 | - |
| TCGA-14-1459 | 2 | - |
| TCGA-16-1063 | 2 | - |
| TCGA-16-1460 | 2 | - |
| TCGA-14-1454 | 2 | - |
| TCGA-06-0145 | 2 | - |
| TCGA-06-0128 | 2 | - |
